# Searching for Tissue-Specific Expression Pattern-Linked Nucleotides of UGT1A Isoforms

**DOI:** 10.1371/journal.pone.0000396

**Published:** 2007-04-25

**Authors:** Wei Zhang, Wanqing Liu, Federico Innocenti, Mark J. Ratain

**Affiliations:** 1 Section of Hematology/Oncology, Department of Medicine, The University of Chicago, Chicago, Illinois, United States of America; 2 Committee on Clinical Pharmacology and Pharmacogenomics, The University of Chicago, Chicago, Illinois, United States of America; 3 Cancer Research Center, The University of Chicago, Chicago, Illinois, United States of America; Deutsches Krebsforschungszentrum, Germany

## Abstract

UDP-glucuronosyltransferases 1A isoforms belong to a superfamily of microsomal enzymes responsible for glucuronidation of numerous endogenous and exogenous compounds. The nine functional UGT1A isoforms are encoded by a single UGT1A gene locus with multiple first exons. The expression of the UGT1A transcripts was measured by quantitative RT-PCR in 23 normal human tissues. The tissue-specific expression patterns were observed in 13 tissues. To understand the regulation mechanism that is responsible for the tissue-specific expression patterns, we scanned the DNA sequence alignments of the putative promoter regions, exon 1 sequences and intron 1 sequences for those expression-pattern-linked nucleotides. Using one of the expression-pattern-linked nucleotides for livers as an example, we showed that a database comprised of these expression-pattern-linked nucleotides could be used to generate focused hypotheses on the problem of tissue-specific expression, which is critical for tissue-specific pharmacodynamics of anticancer drugs.

## Introduction

Human UDP-glucuronosyltransferase (UGT) 1A is a subfamily of UGT enzymes that glucuronidate xeno-/endobiotics and many other substrates such as steroids and bilirubin to make the metabolic products more easily excreted from the body via the urinary and biliary tracts [1], [2]. At least 12 UGT1A isoforms have been identified [3]. Nucleic acid sequence analysis indicates that UGT1A2, UGT1A11, and UGT1A12 encode pseudogenes [3].The nine functional UGT1A isoforms (UGT1A1, UGT1A3, UGT1A4, UGT1A5, UGT1A6, UGT1A7, UGT1A8, UGT1A9, UGT1A10) are encoded by a single UGT1A gene locus with multiple first exons, located on chromosome 2q37 [4], [5]. Located in the 3′ region of the locus are exons 2–5, which encode the conserved 245 amino acids of the carboxyl region. Each first exon is flanked by polymerase II recognition sequences. Meanwhile, the splicing out of each intron fit the rule of GT-AG on the exon-intron boundaries. These suggest that each gene may be individually regulated [4], [6].

Reports regarding UGT1A mRNA expression profiles indicate that each tissue contains a selective complement of UGT1A gene products [7]. Though not extensively studied to date, differential expression of UGT1A isoforms has been observed in hepatic and extrahepatic tissues [6], [8]. As UGT1A genes play critical roles in the metabolism of xeno-/endobiotics, their tissue-specific expression would be very important to organ/tissue-specific toxicity and response to variety of drugs. Therefore, understanding the mechanism underlying the tissue-specific expression of these genes would be essential to the pharmacogenetics and pharmacodynamics of drugs to be glucuronidated. However, the precise distribution and expression of the entire UGT1A locus in human tissues have not been systematically examined. In the present work, quantitative real-time polymerase chain reaction (RT-PCR) was used to detect transcripts of all of the nine functional members of the UGT1A locus in 23 normal human tissues. Unique tissue-specific expression patterns were observed in 13 tissues. Since individual regulation of the unique UGT1A transcripts has not been conclusively demonstrated and often hard to test experimentally, we applied an *in silico* approach to search for the expression-pattern-linked nucleotides. Due to the unique structure of UGT1A locus, specific nucleotides in promoter regions, exon 1 sequences, intron 1 sequences may (jointly) contribute to the observed tissue-specific expression patterns. Some possible mechanisms include promoter efficiency through transcription factor binding and alternative or false splicing. Specifically, we analyzed the putative promoter regions, exon 1 sequences and intron 1 sequences for nucleotides associated with tissue-specific expression, focusing on the eleven tissues that had more than two expressed UGT1A isoforms. The resulting database or pool of the expression-pattern-linked nucleotides then could be used to generate focused hypotheses on the regulation of the tissue-specific expression of UGT1A isoforms.

## Results and Discussion

**Figure 1:**
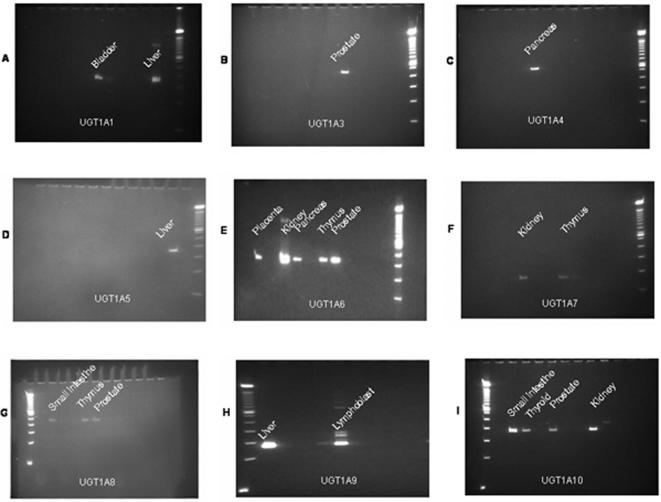
Expression of UGT1A isoforms among multiple human tissue samples. For each functional UGT1A isoform (A through I), an example for the PCR gels for expression in multiple human tissues together with molecular weight markers is shown.

We measured the expression levels of the nine functional UGT1A isoforms in 23 normal tissues. UGT1A genes were not expressed in 10 tissues (brain, skeletal muscle, spleen, uterus, mammary gland, pituitary body, bone marrow, lymph node, leukocyte and all blood fractions), while the tissue-specific expression patterns were observed in other 13 tissues, among which placenta and lung had only one expressed isoform (UGT1A6) (Table 1). Some examples of the expression of UGT1A isoforms among multiple human tissue samples are provided in Figure 1. The relative expression levels based on the density of PCR products of the isoforms in each tissue are shown in Table 1. Some published tissue-specific expression patterns have been confirmed in our RT-PCR expression data. For examples, our data confirmed that UGT1A1, UGT1A3, UGT1A4, UGT1A6 and UGT1A9 are expressed in livers [3], [9]–[12], UGT1A6 is expressed in lungs [13], UGT1A7 is expressed in kidneys [13], and UGT1A10 is expressed in intestines [7].

**Table 1:** Relative expression levels of UGT1A isoforms among multiple human tissue samples.

	Placenta	Kidney	Pancreas	Thymus	Prostate	Testis	Small intestine	Thyroid	Bladder	Lymphoblast	Tonsil	Liver	Lung
UGT1A1			1			1	1		3			3	
UGT1A3					4							10	
UGT1A4			4									11	
UGT1A5				4								2	
UGT1A6	3	7	2	2	3			1	6		3	4	3
UGT1A7		2		1				1	1	1	3		
UGT1A8				1	1		1		3				
UGT1A9		5	1	1	1	1	1			3		4	
UGT1A10		3			1		3	1	3	1	3		

UGT1A isoforms showed specific expression pattern in different tissues. For example, in livers, only 6 UGT1A isoforms (UGT1A1, UGT1A3, UGT1A4, UGT1A5, UGT1A6, UGT1A9) had expression detected at different levels. Our major aim was to explore an approach to study the mechanism responsible for the observed tissue-specific expression patterns. Due to the unique structure of the UGT1A cluster, we hypothesized that the nucleotides that were linked to the expression patterns might (jointly) contribute to the regulation of expression in different tissues. A database or pool of expression-pattern-linked nucleotides was constructed by scanning the putative promoters, intron 1 sequences as well as exon 1 sequences of the nine functional UGT1A isoforms. The CLUSTAL W[14]-generated multiple alignments were used to identify those specific nucleotides that had the same pattern with the expression in a particular tissue. For example, to search for nucleotides that may be linked to the expression patterns of UGT1A isoforms in livers, we scanned the multiple alignments in promoters (exon 1 sequences, intron 1 sequences) for those nucleotides that were identical in UGT1A1, UGT1A3, UGT1A4, UGT1A5, UGT1A6 and UGT1A9 (isoforms expressed in livers, Table 1), but different in UGT1A7, UGT1A8 and UGT1A10 (isoforms not expressed in livers, Table 1). The complete database including the original nucleotide sequences, multiple alignments and the identified nucleotides for each tissue are provided in the supplemental materials. The database then could be used to generate focused hypotheses that could be tested further experimentally and/or bioinformatically.

To show an example, we used the Match [15] program to search the TRANSFAC database [16] for potential TFBS around one of the expression-pattern-linked nucleotides for livers. The liver-expression-specific nucleotide closest to the TSS is a C at the 3028^th^ bp in the multiple alignment of 3447 bp of the putative promoters for the six UGT1A isoforms with hepatic expression and either a gap or a G for the three UGT1A isoforms not expressed in liver (see supplemental materials). The Match program identified a TFBS for AP-1 (activator protein 1) for three of the six hepatically-expressed UGT1A isoforms (UGT1A3, UGT1A4, UGT1A5). A testable hypothesis then could be that the binding site for AP-1 close to the TSS may be contributing to the expression in liver for these three isoforms, but not the other three hepatically-expressed isoforms. Of course, this doe not necessarily mean that this particular transcription factor is the only determinant for hepatic expression. The example just showed that we could now use the database to prioritize our efforts and guide further studies based on these specific nucleotides. Besides promoter efficiency, other mechanisms such as alternative splicing may also contribute to the observed tissue-specific expression patterns. To generate such focused hypotheses, we could search the tissue-specific nucleotides in our database for potential exonic or intronic splicing enhancers [17], [18]. Therefore, the identification of these expression pattern-linked nucleotides in different tissues provided potential working targets, both experimentally and/or bioinformatically, for testing and generating focused hypotheses on the mechanisms responsible for the tissue-specific expression of UGT1A isoforms.

### Footnotes

The supplemental materials can be accessed at http://home.uchicago.edu/∼wzhang1/PONE/UGT1A/and will be deposited into PharmGKB (http://www.PharmGKB.org).

## Materials and Methods

### Human normal tissue samples

Complementary DNA (cDNA) from a total of 23 human normal tissues was purchased from Clontech (Clontech Laboratories, Inc., Palo Alto, CA, USA). These tissues include human brain, placenta, skeletal muscle, kidney, pancreas, spleen, thymus, prostate, testis, ovary, small intestine, uterus, mammary gland, thyroid, pituitary body, bone marrow, bladder, tonsil, lymph node, leukocyte, blood fractions, liver and lung.

### Expression of UGT1A isoforms in normal tissues

The expression of the UGT1A transcripts was measured in the 23 human normal tissues by PCR using TITANIUM Taq DNA Polymerase (Clontech). Briefly, PCR was set up in a 50 µl vol reaction with 3 µl of cDNA as template. Primers for each exon 1 were 5′-aacaaggagctcatggcctcc-3′ (UGT1A1), 5′-tgttgaacaatatgtctttggtcta-3′ (UGT1A3), 5′-gaaggaatttgatcgcgttac-3′ (UGT1A4), 5′-ggtggtggtcctcaccctg-3′ (UGT1A5), 5′-cagctgtcctcaagagagatgtgga-3′ (UGT1A6), 5′-gttgcgaactgactttgttttggag-3′ (UGT1A7), 5′-ggtcttcgccaggggaatagg-3′ (UGT1A8), 5′-ttctccaaacacctgttacggag-3′ (UGT1A9), 5′-cctctttcctatgtccccaatga-3′ (UGT1A10). The reverse primer (5′-ccaatgaagaccatgttgggc-3′) was shared by all UGT1A genes. PCR reactions were denatured initially at 95°C for 1 min, and then cycled 35 times at 95°C for 30s, annealing and extension at 65°C for 3 min. GAPDH gene was amplified with same conditions as above by using primers 5′-tgaaggtcggagtcaacggatttggt-3′ and 5′-catgtgggccatgaggtccaccac-3′ and served as an internal control for cDNA quantity and quality.

### DNA sequences of UGT1A isoforms

The GenBank/NCBI reference sequence for human UDP-glucuronosyltransferase 1 family, polypeptide A cluster on chromosome 2 (NG_002601) was used to retrieve the following regions for the UGT1A isoforms.

- Promoter regions; The putative promoter regions of the nine functional UGT1A isoforms are defined as the sequences of 1-3000 bp upstream of the transcription start sites (TSS).
- Intron 1 sequences; Because the shortest intron 1 (UGT1A1's) is less than 6 kb and the 3′ ends of the intron 1 sequences are shared among the isoforms, we scanned the 5 kb segments immediately downstream of the first exons of the nine functional UGT1A isoforms.
- Exon 1 sequences: The exon 1 sequences of the nine functional UGT1A isoforms are distinct, so they were included in the analysis.

### Identification of expression-pattern-linked nucleotides

The nucleotide sequences of the UGT1A isoforms were aligned using CLUSTAL W [14] (default settings) at the European Bioinformatics Institute web site (http://www.ebi.ac.uk/clustalw/). The alignments and the original DNA sequences are provided in the supplemental materials. The multiple alignments were then scanned for those nucleotides that were linked to a particular expression pattern for each isoform and each tissue type. Therefore, the expression-pattern-linked nucleotides are in the form of an ordered vector *N_t_* = [n_1_(p_1_),n_2_(p_2_),…n_j_(p_j_)], where *N_t_* represents the vector of specific nucleotides for tissue t, n is a particular nucleotide, p is an integer for the position in an alignment. The elements of *N_t_* are [n_j_(p_j_):ψ(p_j_)], where ψ represents the set of conserved nucleotides of UGT1A isoforms with expression in a particular tissue at a particular position p. The identified nucleotides and their flanking sites (25 bp upstream/downstream) were then output as entries of the database.

### Prediction of transcription factor binding sites using Match

To show an example that the database comprised of expression-pattern-linked nucleotides could be used to generate focused hypotheses on tissue-specific expression, we used the Match [15] program at the Gene Regulation web site (http://www.gene-regulation.com/) to predict the transcription factor binding sites (TFBS) in the regions that contain one of the liver-specific nucleotides. The Match program searches the TRANSFAC database [16] for potential TFBS (core cutoff = 0.95, matrix cutoff = 0.90) using high quality database for vertebrates [15]. The search results (TFBS) that cover the expression-pattern-linked nucleotide were compared with the expression patterns of the UGT1A isoforms.
